# Evaluating the B-cell C3d:CR2 innate-adaptive immune interaction as a therapeutic target in lupus

**DOI:** 10.1186/ar3972

**Published:** 2012-09-27

**Authors:** VM Holers, JM Thurman, JP Hannan, L Kulik

**Affiliations:** 1 University of Colorado School of Medicine, Aurora, CO, USA

## Background

B-cell targeted therapies are important strategies in human systemic lupus erythematosus (SLE). Previous studies have shown that B-cell complement receptor type 2 (CR2/CD21), along with its C3 activation fragment antigen-bound ligand designated C3d, play essential roles in the innate-adaptive immune interface and development of antibodies to foreign antigens. CR2 acts with CD19 to greatly amplify B-cell receptor signals. We hypothesize that a similar role is played by this receptor-ligand pair in the development of high-affinity IgG autoantibodies in patients with SLE. Prior gene-targeting studies have suggested, however, that CR2 expression may be needed to maintain tolerance. These studies are confounded, however, because not only is CR2 absent but due to murine-specific gene structures another receptor designated CR1 is also deleted at the same time. CR1 is a receptor for complement fragment C4b, whose deficiency in humans and murine models leads to lupus. In addition, a recent report of the first identified human CR2-deficient individual revealed a humoral immunodeficiency and not an autoimmune phenotype. Other recent studies have shown in MRL/*lpr *and (NZB×NZW)F1 mice that the use of soluble CR2 as a potential dominant negative inhibitor led to a substantial decrease in autoantibody titers.

## Methods

To address our hypothesis, we developed novel mAbs that disrupt the CR2-C3d interface alone, without affecting the interactions of CR1 with C4b. We immunized *C3^-/- ^*mice with recombinant human C3d, and *Cr2^-/- ^*mice with recombinant murine CR2.

## Results

The resultant human C3d-reactive mAbs inhibited C3d-CR2 binding, did not recognize intact C3/C3b, and cross-reacted with mouse C3d. Two anti-C3d mAbs, 3d29 and 3d8b, along with control mAb were pre-injected into mice before sheep red blood cell (SRBC) immunization. IgG1 responses to SRBC antigen were substantially decreased, consistent with the interruption *in vivo *of C3d binding to CR2. One resulting anti-CR2 mAb (4B2, IgG_1_), which directly blocks binding of C3d to CR2, was injected in wild-type mice and demonstrated no B-cell depletion but maintenance of blockade of CR2 on the B-cell surface for at least 1 month. SRBC immunization of mice pre-injected with mAb 4B2 revealed reduced anti-SRBC levels to levels found in immunized *Cr2*^-/- ^mice. No anti-idiotype antibodies were detected.

## Conclusion

We have developed unique tools to characterize in mouse models of human lupus the pathogenic roles of both the C3d ligand and CR2 components of the CR2-C3d interaction pair.

